# There is a cycle to cycle variation in ovarian response and pre-hCG serum progesterone level: an analysis of 244 consecutive IVF cycles

**DOI:** 10.1038/s41598-020-72597-0

**Published:** 2020-09-25

**Authors:** Sule Yildiz, Kayhan Yakin, Baris Ata, Ozgur Oktem

**Affiliations:** The Division of Reproductive Endocrinology and Infertility, Department of Obstetrics and Gynaecology, Koc University School of Medicine, Davutpasa Cad. No:4, 34010 Topkapi Istanbul, Turkey

**Keywords:** Endocrinology, Medical research

## Abstract

We aimed to answer one key question, that was not previously addressed as to whether serum progesterone (P_4-hCG_ day) and its co-variates (estradiol (E_2-hCG day_) and the number of retrieved oocytes) of a given cycle can be predictive of the subsequent cycle when both cycles are consecutive and comparable for the stimulation protocol, gonadotropin dose and duration of stimulation. We analyzed such 244 consecutive (< 6 months) IVF cycles in 122 patients with GnRH agonist long protocol and found that P_4_, E_2_ and the number of retrieved oocytes significantly vary between the two cycles. Although P_4_ increased (ranging from 4.7 to 266.7%) in the 2nd cycle in 61 patients, E_2_ and the number of retrieved oocytes, which are normally positively correlated with P_4_ paradoxically decreased in the 41% and 37.7% respectively, of these same 61 patients. When a similar analysis was done in the 54 out of 122 patients (44.3%) in whom serum P_4_ was decreased in the 2nd cycle, the mean decrease in P_4_ was − 34.1 ± 23.3% ranging from − 5.26 to − 90.1%. E_2_ and the number of retrieved oocytes paradoxically increased in the 42.3% and 40.7% of these 54 patients respectively. P_4_ remained the same only in the 7 (5.7%) of these 122 patients. These findings indicate that late follicular phase serum P_4_ may change unpredictably in the subsequent IVF cycle. The changes are not always necessarily proportional with ovarian response of previous cycle suggesting that growth characteristics and steroidogenic activities of antral cohorts may exhibit considerable cycle to cycle variations.

## Introduction

Serum progesterone (P_4_) level may elevate during late follicular phase of ovarian stimulation before ovulation is triggered and is considered a negative predictive factor for clinical outcome in assisted reproduction in both GnRH agonist and GnRH antagonist protocols^1^. It is not a true luteinization event because the elevations in serum P_4_ level occurs prior to hCG administration and is not associated with any premature LH surge. This phenomenon is generally observed in stimulated IVF cycles and shows significant correlation with the intensity of ovarian stimulation; hence patients with more growing follicles and retrieved oocytes have higher P levels^1–7^. Interestingly, it was also documented that pre-ovulatory P_4_ elevations may also occur in up to 28% of natural cycles and reduce pregnancy rates. However, the mechanism of P_4_ rise in natural cycles is distinct from stimulated IVF cycles because there is no gonadotropin stimulation or multiple follicle development in the former^8^. In agreement with the close link between the degree of ovarian stimulation with gonadotropins and serum pre-hCG P_4_ level in stimulated IVF cycles, we recently provided a molecular explanation for this phenomenon by showing that FSH stimulation itself promotes P_4_ synthesis and output from human granulosa cells without luteinization by up-regulating the expression and enzymatic activity of the enzyme 3β-hydroxy steroid dehydrogenase (3β-HSD), which converts pregnenolone to progesterone^9^. Therefore, it is likely that pre-ovulatory P_4_ elevation is caused by the insufficiency of the ovary in handling the increased output of precursor steroids generated during multi-follicular development in FSH stimulated IVF cycles. To date, several clinical studies and meta-analyses showed a negative impact of P_4_ elevations before ovulation trigger on the chance of pregnancy in fresh embryo transfer IVF cycles^1,3,4,10^.

In most of the cases in stimulated IVF cycles, the risk for an elevation in serum P_4_ level at late follicular phase before ovulation trigger is closely related to the magnitude of ovarian stimulation, that is also reflected by E_2_ level on the hCG day, and the number of growing follicles > 14 mm on day 10 of stimulation and total and mature oocytes retrieved^11,12^. Therefore, there are significant positive associations between P_4_ and these co-variates. However, one key question remains to be answered as to whether we should expect to see similar pre-hCG serum P_4_ levels and its co-variates in two consecutive IVF cycles comparable for gonadotropins, GnRH analog used, and duration of stimulation. We aimed to answer this question in this non-interventional, retrospective cohort data from a single center.

## Material and methods

### Study design

This study is a retrospective cohort analysis from a single center. It was approved by the Institutional Review Board (IRB) of Koc University (2015.206.IRB2.076). It is not a research study that involve human participation. Therefore, the need of the written informed consent was waived by the Institutional Review Board (IRB) of Koc University. All methods were performed in accordance with the relevant guidelines and regulations of the Institution. We reviewed the electronic database of 8724 IVF cycles that had been performed from 2008 to 2015 in Assisted Reproduction Unit, American Hospital of Istanbul, Turkey. A total of 122 women who had 244 consecutive IVF cycles within a 6-month interval following an unsuccessful cycle, using exactly the same ovarian stimulation protocol in both cycles were identified and included in this study. Patients who underwent stimulation more than 6 months apart or had ovarian surgery, systemic disease that could affect ovarian response to stimulation were excluded.

### Ovarian stimulation and ovulation trigger

Pituitary down-regulation was induced with GnRH agonist leuprolide acetate started 7 days prior to the anticipated day of menstrual bleeding and continued until the day of hCG trigger. Recombinant FSH was started on cycle day three at a dose of 225–300 IU depending upon age, antral follicle count, anticipated or documented previous ovarian response, and body mass index. Ovulation was triggered with 250 µg recombinant hCG (Ovitrelle; Merck-Serono, Istanbul, Turkey) when a leading follicle of ≥ 19 mm and two or more trailing follicles of ≥ 17 mm were recorded. Follicular aspiration was performed 36 h after ovulation trigger. Oocyte retrieval was performed under general anaesthesia using a double lumen needle (Cook Ireland Ltd., Limerick, Ireland) as we described previously^11^.

### Hormone assays

Serum samples for hormone assays were obtained by veni-puncture and assessed using a validated electrochemiluminescence immunoassay (ECLIA method, Cobas 6000, Roche, Basel, Switzerland) as described previously^11^. Analytical sensitivity (lower detection limit) for P_4_ was 0.095 nmol/L (0.030 ng/mL) and the functional sensitivity (defined as lowest analyte concentration that can be reproducibly measured with a between-run coefficient of variation [CV] of < 20%) was 0.48 nmol/L (0.15 ng/mL). The day-to-day CV was 2.9% at 2.31 nmol/L (0.73 ng/mL), 1.4% at 9.57 nmol/L (3.1 ng/mL), and 0.9% at 103 nmol/L (32.4 ng/mL). Analytical sensitivity for E_2_ was 18.4 pmol/L (5 pg/mL). The day-to-day CV for E2 was 6.7% at 27.4 pg/mL, 1.1% at 1270 pg/mL, and 1.9% at 2720 pg/ml. The same assay was used during the study period and was calibrated whenever a new reactive batch was used or whenever an outcome outside the normal range was observed.

### Statistical analysis

Continuous variables in the baseline demographic and IVF characteristics were expressed as mean (SD) or median (25^th^–75th percentile) depending on distribution characteristics. Two-tailed Pearson correlation test and linear regression analysis were used to identify the confounding variables that show significant association with serum P_4_ level. Continuous variables were compared between the groups with paired samples t- test or Wilcoxon signed rank test as appropriate. The significance level was set at 5% (P < 0.05). Graphpad Prism (version 7) and SPSS statistical programs (version 23) were used to analyse the data and create the figures^11^.

## Results

**Figure 1:**
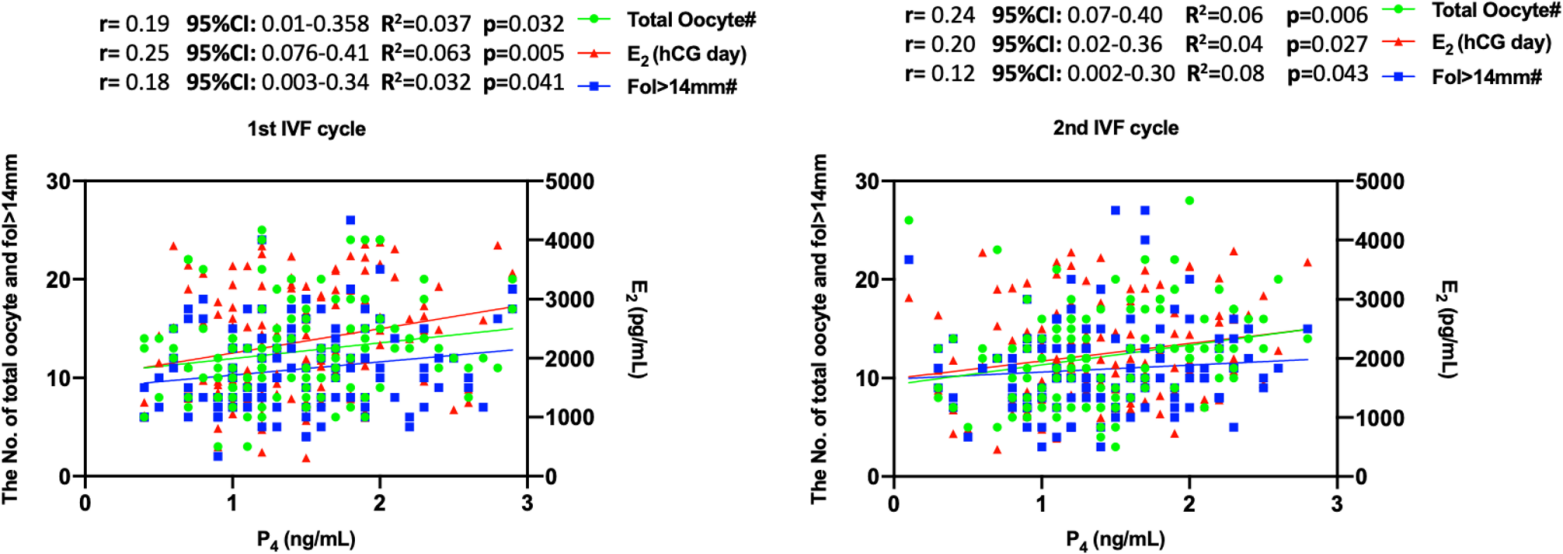
The association of P_4_ level on the hCG day with E_2_ level on the hCG day, and the numbers of fol > 14 mm and retrieved oocytes on the correlation and linear regression analyses in the 1st and 2nd IVF cycles.

Baseline demographic and IVF characteristics of the two consecutive cycles are summarized in Table 1. Male factor infertility (78.5%) was the major indication followed by unexplained infertility (9.4%), tubal factor (11.9), and ovulatory pathologies (1.6%). Average age, daily and total doses of gonadotropins and duration of stimulation, serum levels of P_4_ and E_2_ levels on the hCG day, the numbers of fol > 14 mm on day 10 of stimulation and total and mature oocytes retrieved were similar between the two cycles (Table 1). In both cycles, serum P_4_ level on the hCG day was significantly associated with serum E_2_ level on the hCG day, and the numbers of growing follicles (fol > 14 mm) on day 10 of stimulation, and the oocytes retrieved on the correlation and linear regression analyses (Fig. 1). However, the level of significance was not the same for all three co-variates of P_4_. E_2_ on the hCG day and total oocyte counts appeared to be more closely associated with P_4_ in both 1st and 2nd IVF cycles than the number of fol > 14 mm.

**Table 1:** Baseline demographic and IVF characteristics of the two consecutive IVF cycles.

	1st IVF cycle	2nd IVF cycle	P
Age	31.2 ± 4.6	31.2 ± 4.6	0.6
Daily FSH dose	232.9 (225–300)	231.1 (225–300)	0.08
Total gonadotropin dose^a^	2475 (2250–3000)	2625 (2175–3300)	0.25
Duration of stimulation^a^	10 (9–11)	10 (9–11)	0.5
Serum E_2_ on the hCG day	2270 ± 945	2076 ± 842	0.06
Serum P_4_ on the hCG day	1.44 ± 0.57	1.4 ± 0.57	0.52
The number of fol > 14 mm on day 10	10.9 ± 4.3	10.9 ± 4.4	1
The number of total oocytes	12.6 ± 4.75	12.1 ± 4.68	0.27
The number of mature oocytes	9.19 ± 3.82	8.83 ± 3.53	0.37
The number of metaphase 2 oocytes^a^	9 (7–11)	8 (6–11)	0.47
The number of 2PN embryos^a^	6 (5–8)	6 (5–8)	0.65

Both cycles were comparable for IVF characteristics.

Paired t-test or Wilcoxon signed rank test where appropriate.

^a^Values are median (25th–75th percentile) or n (%).

**Figure 2:**
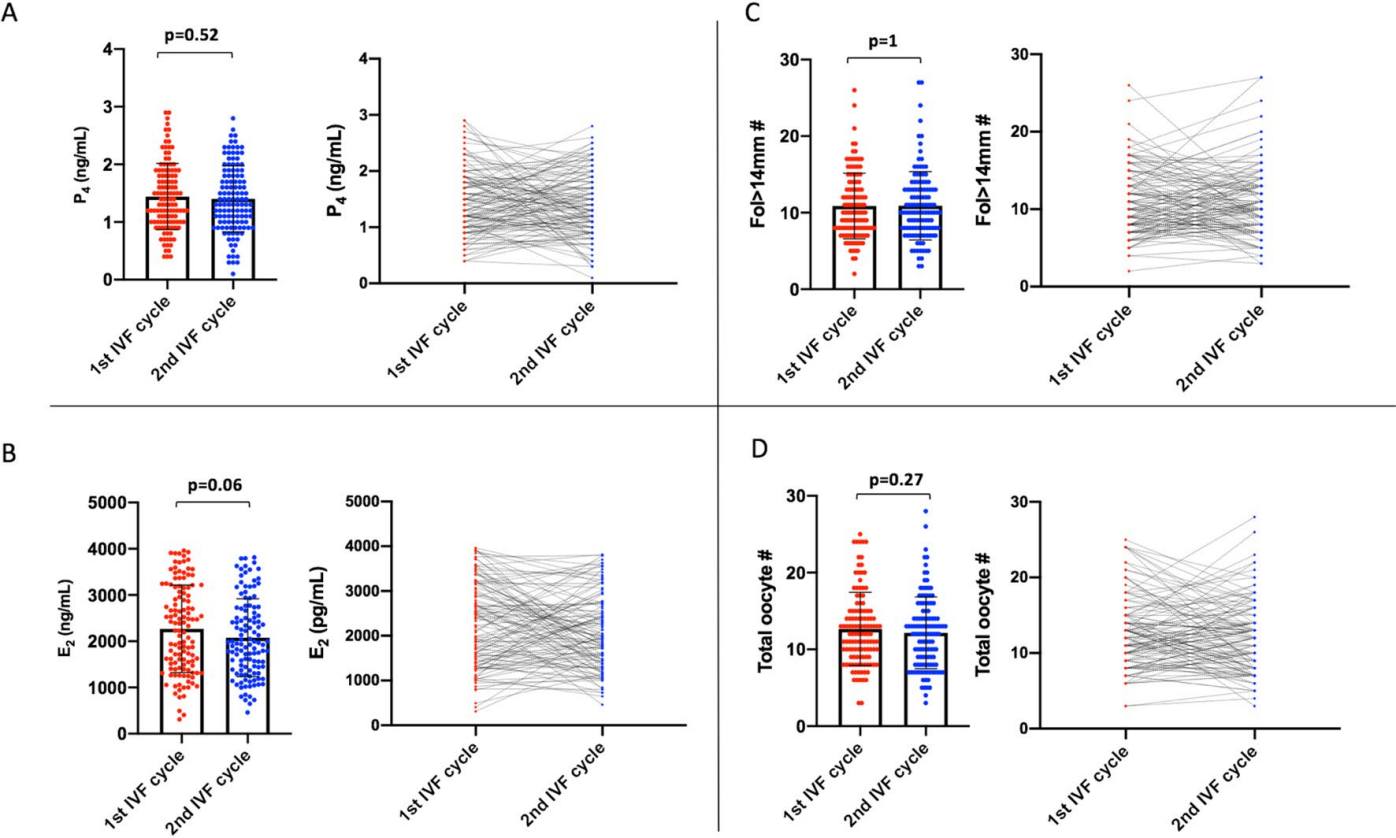
Comparison of the means of P_4_ on the hCG day (**A**) and its co-variates E_2_ on the hCG day (**B**), and the numbers of fol > 14 mm on day 10 of stimulation (**C**) and retrieved oocytes (**D**) between the 1st and 2nd IVF cycles are shown as scatter plot with bars (the left images graphics). The variations in these parameters between the cycles are also shown as lines (the right graphics).

The mean values of P_4_ and its co-variates (serum E_2_ level on the hCG day, and the numbers of growing follicles (fol > 14 mm) on day 10 of stimulation, and the oocytes retrieved) were comparable between the cycles. However, we noticed there were substantial cycle to cycle variations in these markers (Fig. 2).

**Figure 3:**
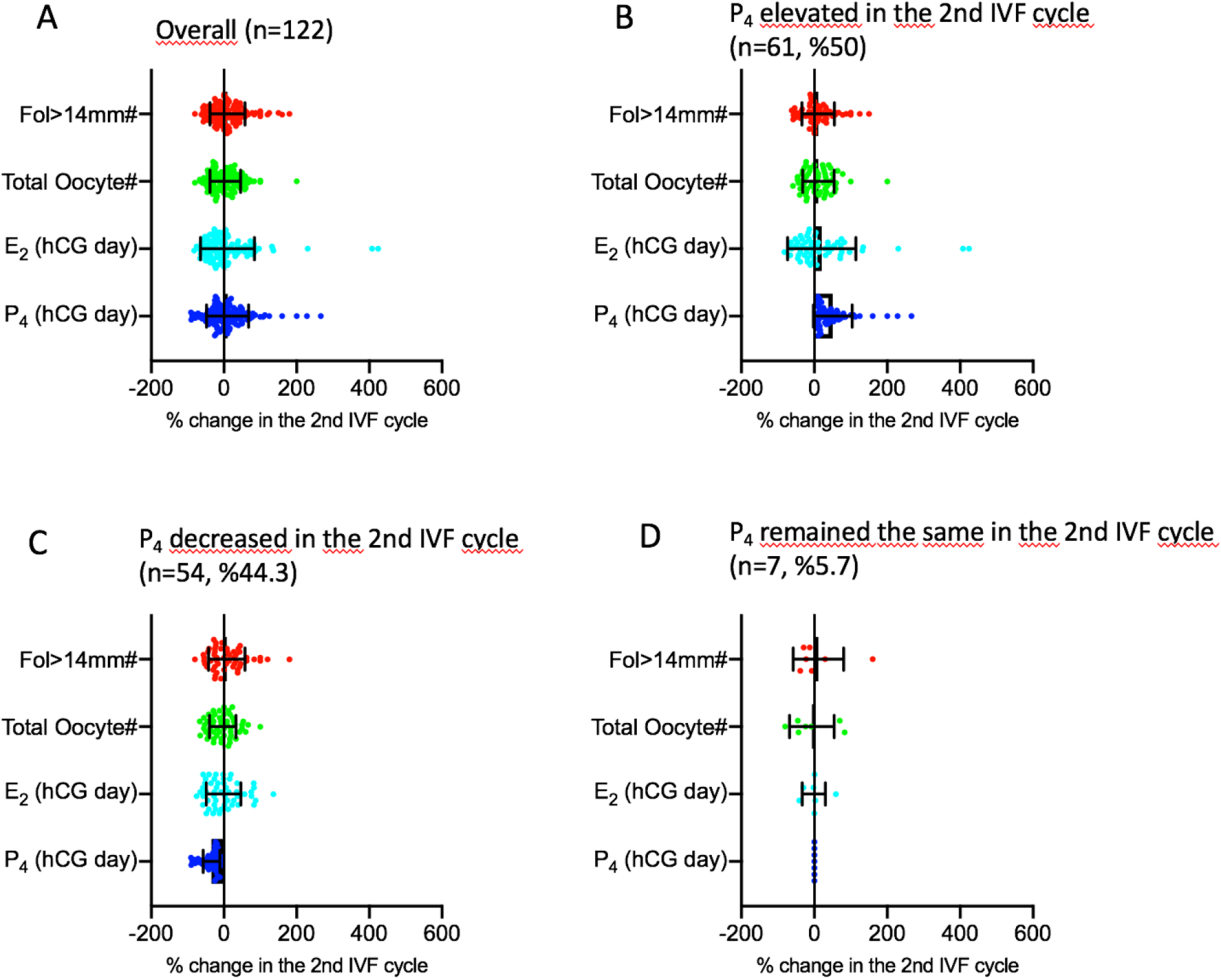
Scatter plot diagrams of the percent changes of P_4_ on the hCG day and its correlates E_2_ on the hCG day, and the numbers of fol > 14 mm on day 10 of stimulation and retrieved oocytes overall (**A**), and when P_4_ in the second cycle is > 0 (**B**), < 0 (**C**) or = 0 (**D**). Paradoxical changes in the co-variates of P_4_ (Y-axis) when P_4_ increased and decreased are visible on the X-axis.

Next, we investigated how serum P_4_ level and its correlates changed in the 2nd IVF cycle in comparison to their corresponding 1st cycle values in each patient. This was expressed as the percentage of change based on the formula as follows: [(P_4 in the 2nd cyle _− P_4 in the 1st cyle_) × 100/P_4 in the 1st cyle_)]. We observed that there are indeed significant variations in the 2nd cycle when compared to their corresponding 1st cycle values of P_4_ (%change: − 90.1 to 266.7%), E_2_ (− 81.7 to 424%), Fol > 14 mm (− 80 to 180%) and oocyte number (− 80 to 200%). The mean, median and percentiles of the percent changes for each of these variables are shown in the Table 2 and depicted as a scatter plot diagram in the Fig. 3A. Then, the IVF cycles were categorized into three groups according to the percent change of P_4_ in the second IVF cycle as being > 0; = 0 and < 0 in order to investigate how the co-variates of P_4_ (E_2-hCG day_, fol > 14 mm and retrieved oocyte counts) changed in relation to a particular change in P_4_. By doing so we aimed to identify the cycles in which the co-variates of P_4_ exhibited paradoxical rather than parallel changes with P_4._ in the subsequent cycles in comparison to the first cycles.

**Table 2:** Descriptive summary statistics of the percent changes of P_4_ on the hCG day and its correlates E_2_ on the hCG day, and the numbers of fol > 14 mm on day 10 of stimulation and retrieved oocytes.

	% change in the 2nd IVF cycle			
	P_4_	E_2_	Fol > 14 mm#	Total oocyte#
Mean	10.2	9.78	9.58	3.73
Median	2.38	− 4.9	0.0	0.0
Std. deviation	57.9	74.2	48	41.8
Minimum	− 90.1	− 81.7	− 80.0	− 80.0
Maximum	266.7	424	180.0	200.0
25 Percentiles	− 25.0	− 33.9	− 23	− 28.0
50 Percentiles	2.38	− 4.9	0.0	0.0
75 Percentiles	33	36.4	33.3	30.0

**Figure 4:**
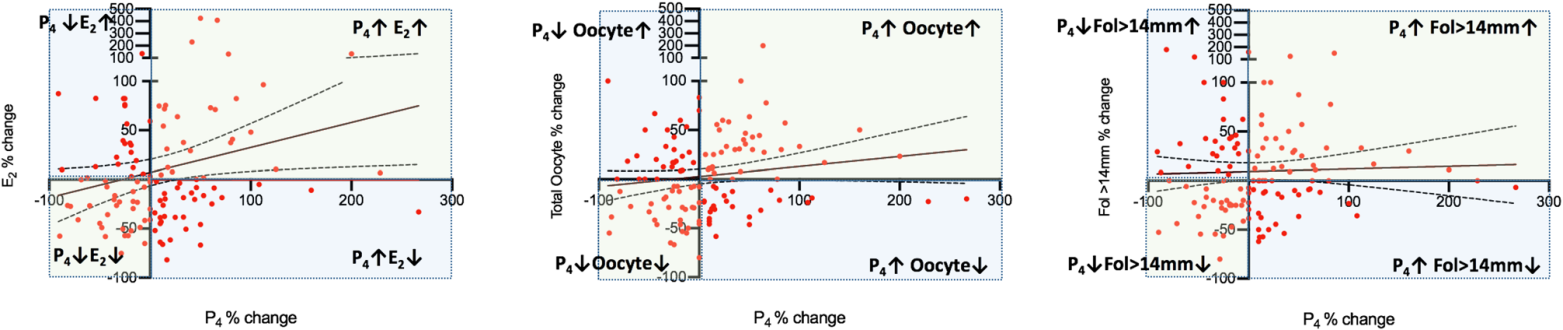
Histogram depiction of the percent change of P_4_ in the 2nd cycle (x-axis) and the corresponding percent change in E_2_, and the numbers of fol > 14 mm and retrieved oocytes (y-axis). Light green areas show the cycles in which there are parallel changes in P_4_ and its co-variates (E_2_ and the numbers of fol > 14 mm and retrieved oocytes). Light blue areas demonstrate the cycles in which there were paradoxical changes in P_4_ and its correlates. Solid line: linear regression, dotted line 95% confidence intervals.

According to the categorization described above serum P_4_ level on the hCG day was higher in the 2nd IVF cycle than their corresponding 1st cycle levels in 61 of 122 patients (50.0%); remained unchanged in the seven (5.7%) and decreased in the 54 patients (44.3%). The mean increase in P_4_ in the 2nd cycle was 50.6 ± 53.3% ranging from 4.76 to 266.6%. However, the mean and range of changes in the co-variates of P_4_ were 20.3% (− 81.8 to 424.6%) for E_2_; 10.9% (− 61.9 to 150%) for fol > 14 mm; and 11.2% (− 58.3 to 200%) for total oocyte numbers, suggesting that not all elevations in P_4_ are accompanied by parallel increases in its-covariates in the 2nd IVF cycle (Table 3, Fig. 3B). We found that E_2_ and the numbers of fol > 14 mm and retrieved oocytes paradoxically decreased in the 55%, 37.7% and 41% respectively, of these 61 patients despite the elevations in P_4_ levels in their 2nd IVF cycle. The percent decreases ranged from − 2.03 to − 81.8 for E_2_; from − 6.67 to − 61.9 for Fol > 14 mm; and from − 7.14 to − 58.3 for the oocyte count. The distribution of the parallel and paradoxical changes in the co-variates of P_4_ in relation to the changes in P_4_ are depicted as a histogram in the Fig. 4.

**Table 3:** Descriptive summary statistics of the percent changes of P_4_ in the 2nd cycle > 0.

	% change P_4_ > 0 in the 2nd IVF cycle			
	P_4_	E_2_	Fol > 14 mm#	Total oocyte#
Mean	50.6	20.3	10.9	11.2
Median	33.3	− 6.5	0.0	10
Std. deviation	53.3	93.5	44.4	43.2
Minimum	4.76	− 81.8	− 61.9	− 58.3
Maximum	266.7	424.6	150.0	200.0
25 Percentiles	14.2	− 33.7	− 12.9	− 22.8
50 Percentiles	33.3	− 6.57	0.0	10
75 Percentiles	64.1	46.2	33.3	34.5

Despite the increases in P_4_, the percent changes in its co-variates exhibited significant variations.

When a similar analysis was done in the 54 out of 122 patients (44.3%) in whom serum P_4_ was decreased in the 2nd cycle, the mean decrease in P_4_ was  − 34.1 ± 23.3% ranging from − 5.26 to − 90.1% (Table 4). It appeared that E_2_ and the numbers of fol > 14 mm and retrieved oocytes paradoxically increased in the 42.3%, 45.3% and 40.7% of these 54 patients respectively. The percent increases ranged from 1.60 to 136.3% for E_2_; from 6.25 to 180 for fol > 14 mm; and from 8.3 to 100 for the oocyte count (Figs. 3C and 4).

**Table 4:** Descriptive summary statistics of the percent changes of P_4_ in the 2nd cycle < 0.

	% Change P_4_ < 0 in the 2nd IVF cycle			
	P_4_	E_2_	Fol > 14 mm#	Total oocyte#
Mean	− 34.1	− 0.94	7.75	− 3.45
Median	− 26.4	− 7.47	0.0	− 7.1
Std. deviation	23.3	47.4	49.9	36.5
Minimum	− 90.9	− 75.2	− 80.0	− 66.6
Maximum	− 5.26	136.3	180.0	100.0
25 Percentiles	− 45.2	− 39.6	− 25.0	− 29.5
50 Percentiles	− 26.4	− 7.47	0.0	− 7.14
75 Percentiless	− 16.6	26.1	37.5	18.6

Despite the decreases in P_4_, the percent changes in its co-variates exhibited significant variations.

Since the number of patients was too small (n = 7) in the group where serum P_4_ level remain unchanged between the cycles no statistical analysis was conducted (Fig. 3D).

We also investigated if P_4_ elevation in the previous cycle can predict its occurrence in the next cycle because it was shown that history of P_4_ elevation can predict its occurrence again in the next cycle independent of ovarian stimulation^13^. When a cutoff level of 1.5 ng/mL was adopted for serum P_4_ we found that 57 (46.7%) of 122 patients had serum P_4_ level ≥ 1.5 ng/mL in the 1st cycle. And 32 (56.1%) of these 57 patients continued to have P_4_ level exceeding this threshold in the 2nd cycle. Interestingly, despite P_4_ elevations, 14 (43.8%) of these 32 women had paradoxical decreases in the number of retrieved oocytes in their 2nd cycle with the percent decreases ranging from − 7.14 to − 80.0. Similar decreases (− 2.03 to − 65.2%) were observed in the E_2_ level in 21 of these 32 patients (67.7%).

Most of the patient in this cohort 94/122 (77%) were normal responders (4–15 oocytes in the 1st cycle). Therefore we conducted a subgroup analysis analysis for this group and obtained similar results: Serum P_4_ level on the hCG day in the 2nd IVF cycle was higher than their corresponding 1st cycle levels in 48 of 94 patients (51.1%), while it remained unchanged in five (5.3%) and decreased in the 54 patients (43.6%). E_2_ and the numbers of fol > 14 mm and retrieved oocytes paradoxically decreased in the 51.1%, 35.4% and 33.3% of these 48 patients respectively. The percent decreases ranged from − 2.03 to − 81.8 for E_2_; from − 6.67 to − 57.1 for Fol > 14 mm; and from − 7.14 to − 58.3 for the oocyte count (Supplementary data).

## Discussion

We have shown in this study that serum P_4_ level on the hCG day and its co-variates (E_2_ on the hCG day and the numbers of growing follicles > 14 mm on day 10 of stimulation and retrieved oocytes) may exhibit significant cycle to cycle variations even if both cycles are consecutive and comparable for the stimulation protocol, the type and dose of gonadotropin and duration of stimulation. The increase in serum P_4_ level in the 2nd cycle was not always associated with parallel increases in its co-variates. There were some paradoxical inverse changes in these co-variates that were normally supposed to be in a positive association with P_4_. Even though P_4_ was significantly associated with E_2_ and the numbers of fol > 14 mm and retrieved oocytes in each cycle itself, not all decrease or elevations in P_4_ in the subsequent cycle were accompanied by parallel changes in its co-variates. These findings suggest that the growth and steroidogenic characteristics of antral cohorts in response to exogenous FSH may vary and may not reliably be predictive of the next cycle even if both cycles are comparable and successive.

It is unclear why there are differences between two cohort of antral follicles of two different cycles exposed to FSH at the same dose and duration. Previous studies documented that there might be fluctuations in the number of antral follicles and AMH levels at early follicular phase between the cycles^14,15^. Inter-cycle variability might not be confined to cohort of growing antral follicles itself as there could be differences in the expression of FSH receptors of granulosa cells of growing follicles and their response to exogenous FSH. Intraovarian actions of FSH and/or the degree of ovarian stimulation might be responsible for premature P output from granulosa cells without luteinization. Steroidogenic activity of the ovarian follicle changes depending on this developmental stage as well as its receptor abundancy and sensitivity. Ovarian stimulation with exogenous FSH is a continuum of multifollicular development with a number of follicles at different stages of development all contributing to the total steroid synthesis at different levels, any given time-point, such as the day of ovulation triggering^16^. Even when the level of steroidogenesis in each granulosa cell or growing follicle does not increase, total steroid synthesis would increase as a factor of increased number of growing follicles and their steroidogenic granulosa cell mass. Another plausible explanation would be the increase in number or sensitivity of FSH and/or LH receptors on the granulosa cells in response to exogenous gonadotropin stimulation. If there are intrinsic differences among the cohorts of antral follicles recruited by FSH in terms of their FSH receptor expression and responsiveness, their growth kinetics and steroidogenic activity may change from cycle to cycle. A particular P_4_ level at late follicular phase that was reached after achieving a certain magnitude of ovarian response to stimulation by FSH in one IVF cycle might not necessarily produce the same degree of ovarian stimulation and P_4_ levels in the next cycle. Variations in the level of significance for the co-variates of P_4_ between the cycles supports this notion while recognizing at the same time that these differences could be related to the relatively small number of subjects in each cycle.

We have recently provided molecular evidence that FSH itself up-regulates the expression and enzymatic activity of the enzyme 3β-hydroxy steroid dehydrogenase (3β-HSD) and promotes P_4_ output from human granulosa cells and ovarian tissue samples without luteinization^9^. Therefore, serum P_4_ level before ovulation trigger is significantly associated with the markers of the degree of ovarian stimulation in multivariate regression analysis, that are the co-variates of P_4_ and include the number of growing follicles in response to FSH on day 10 of stimulation, E_2_ level on the hCG day and the numbers of oocytes retrieved^17^.

Data regarding variations in the hormonal profile of women undergoing a similar type ovarian stimulation is limited. A retrospective analysis of 197 women with multiple treatment cycles, showed that premature progesterone elevation is likely to recur in repetitive stimulation cycles (OR 8.4; 95% CI 2.8–24.9)^13^. The increased likelihood of recurrence persisted when the regression model was adjusted for the intensity of ovarian stimulation. In the same study, basal P level at the initiation of stimulation was independently associated with P_4_ levels on triggering day. Authors suggested that in the presence of a corpus luteum that had not undergone functional luteolysis might be responsible from high levels of late follicular phase P_4_. It was also suggested that persistent high P_4_ synthesis in repeated cycles might be related with patient-specific intrinsic defects in ovarian or adrenal steroidogenesis^18^. In our study, 32 of 122 women (26.2%) had persistent high (≥ 1.5) P_4_ levels in both cycles. Interestingly, 14 (43.8%) of these women had paradoxical decreases in the number of retrieved oocytes in their 2nd cycle with the percent decreases ranging from − 7.14 to − 80.0. Similar paradoxical decreases (− 2.03 to − 65.2%) were observed in the E_2_ level on the hCG day of the 2nd IVF cycle in 21 of these 32 patients (67.7%). These results provide additional evidence that the co-variates of P_4_ may not reliably predict its elevation again in the subsequent cycle. A recent prospective study reported significant intra-day variations in serum progesterone levels during the day of ovulation trigger (Gonzales-Foruria et al. 2019). P_4_ level was highest early in the morning and then gradually decreased throughout the day^19^. Hormone measurements in the blood samples were performed at the same time period of the day early in the morning between 08:00 and 10:00 AM in our study.

These findings were obtained in a relatively small number of IVF patients most of whom were good responders and male factor infertility was the main indication for IVF in majority of the cases. Currently, it is unclear if these results vary depending upon infertility etiology and the types of ovarian stimulation protocol and ovarian response. This is also true when there are accompanying ovarian pathology or other disease that may alter ovarian response to stimulation such as endometriosis, which is a complex disease with genetic, epigenetic and immunologic aberrations^20–22^. Diminished ovarian reserve or poor response to stimulation are commonly observed in patients with endometriosis undergoing ovulation induction or ovarian stimulation^22,23^. Despite many efforts we still do not have a serum hormone marker or a correct algorithm to choose the optimal starting dose of FSH in patients with low and high ovarian reserve and in those with PCOS and high AMH^24,25^. Apparently, bi-directional communication between the oocyte and cumulus granulosa cells plays role in the response to gonadotropins, ovulation, oocyte maturation and IVF success^26,27^. It is unknown if this bi-directional communication varies from follicle to follicle in a given cycle or between the two consecutive cycles. It also should be remembered that all required steps of controlled ovarian stimulation should be accomplished for best practice in IVF^28^ as there are other key factors in addition to premature P_4_ elevation that might impact the success of IVF such as embryo transfer technique^29^.

To the best of our knowledge this is the first study that analyzed such characteristics of two successive IVF cycles. However, there are several imitations of this study such as its retrospective design, data from a single center, and inclusion of a highly specific group of patients with two consecutive ovarian stimulations cycles within a specified time-period using exactly the same stimulation protocol. Although the design was intended to limit confounding variables like changes in stimulation protocol, dose of gonadotropins and ovarian aging, it limits the number of patients in the study and compromises the generalizability of the findings.

## Conclusion

Serum progesterone (P_4-hCG_ day) and its co-variates (estradiol (E_2-hCG day_) and the numbers of growing follicles > 14 mm and retrieved oocytes) may exhibit significant variations between the two cycles even when both cycles are consecutive and comparable for the stimulation protocol, gonadotropin dose and duration of stimulation. Therefore, the growth characteristics and steroidogenic activities of growing antral cohorts might change from cycle to cycle. The parameters of a previous IVF cycle might not accurately predict the subsequent cycle. These findings need to be confirmed in larger number of IVF patients with different infertility etiologies, ovarian stimulation protocols and ovarian response types.

## Supplementary information

Supplementary file1
