# Supplementary material for: There is a cycle to cycle variation in ovarian response and pre-hCG serum progesterone level: an analysis of 244 consecutive IVF cycles
Source: Sci Rep. 2020 Sep 25;10:15793. doi: 10.1038/s41598-020-72597-0 (PMC7519678; doi:10.1038/s41598-020-72597-0)

**THERE IS A CYCLE TO CYCLE VARIATION IN OVARIAN RESPONSE AND PRE-HCG SERUM PROGESTERONE LEVEL: AN ANALYSIS OF 244 CONSECUTIVE IVF CYCLES**

Sule Yildiz MD, Kayhan Yakın MD, Baris Ata MD, Ozgur Oktem MD,

Koc University School of Medicine Department of Obstetrics and Gynecology, the Division of Reproductive Endocrinology and Infertility, Istanbul, Turkey

**Correspondence:**

Ozgur Oktem MD

Koc University School of Medicine

Department of Obstetrics and Gynecology

The Division of Reproductive Endocrinology and Infertility

Davutpasa Cad. No:4, 34010

Topkapi, Istanbul, Turkey

Tel: + (90) 850 250 8 250

Email: [ooktem@ku.edu.tr](mailto:ooktem@ku.edu.tr)

**SUPPLEMENTARY DATA FOR NORMO-RESPONDERS**

**Supplementary Table-1:** Descriptive summary statistics of the percent changes of P_4_ on the hCG day and its correlates E_2_ on the hCG day, and the numbers of fol>14mm on day 10 of stimulation and retrieved oocytes.

**Supplementary Table-2:** Descriptive summary statistics of the percent changes of P_4_ in the 2^nd^ cycle>0. Despite the increases in P_4_, the percent changes in its correlates exhibited significant variations.

**Supplementary Table-3:** Descriptive summary statistics of the percent changes of P_4_ in the 2^nd^ cycle<0. Despite the decreases in P_4_, the percent changes in its correlates exhibited significant variations.

**Supplementary Figure-1:** Scatter plot diagrams of the percent changes of P_4_ on the hCG day and its correlates E_2_ on the hCG day, and the numbers of fol>14mm on day 10 of stimulation and retrieved oocytes overall (3A), and when P_4_ in the second cycle is >0 (3B), < 0 (3C) or =0 (3D).

**Supplementary Figure-2:** Histogram depiction of the percent change of P_4_ in the 2^nd^ cycle (x-axis) and the corresponding percent change in E_2_, and the number of fol>14mm and retrieved oocytes (y-axis). Normal and paradoxic changes in P_4_ and its correlates are shown.

**Supplementary Table-1**

|  | **% change in the 2^nd^ IVF cycle** | | | |
| --- | --- | --- | --- | --- |
|  | **P_4_** | **E_2_** | **Fol>14mm#** | **Total oocyte#** |
| **Mean** | 9.6 | 8.34 | 5.29 | 2.67 |
| **Median** | 2.38 | -8.24 | 0.00 | 0.00 |
| **Std. Deviation** | 55.6 | 76.2 | 46 | 40.7 |
| **Minimum** | -89.9 | -81.7 | -61.9 | -80 |
| **Maximum** | 266.6 | 424.6 | 180 | 200 |
| **25**  **Percentiles 50**  **75** | -25 | -37.4 | -25 | -27.9 |
|  | 2.38 | -8.24 | 0 | 0.00 |
|  | 33.9 | 34.8 | 28.9 | 27.9 |

**Supplementary Table-2**

|  | **% change P_4_>0 in the 2^nd^ IVF cycle** | | | |
| --- | --- | --- | --- | --- |
|  | **P_4_** | **E_2_** | **Fol>14mm#** | **Total oocyte#** |
| **Mean** | 48.4 | 18.4 | 5.15 | 10.9 |
| **Median** | 33.3 | -8.4 | 0.00 | 10 |
| **Std. Deviation** | 50.8 | 96.4 | 41.3 | 43.2 |
| **Minimum** | 4.76 | -81 | -61.9 | -58.3 |
| **Maximum** | 266.6 | 424 | 150 | 200 |
| **25**  **Percentiles 50**  **75** | 14.2 | -34.8 | -14.2 | -22.2 |
|  | 33.3 | -8.4 | 0.00 | 10 |
|  | 63.6 | 49.6 | 25 | 33.3 |

**Supplementary Table-3**

|  | **% change P_4_<0 in the 2^nd^ IVF cycle** | | | |
| --- | --- | --- | --- | --- |
|  | **P_4_** | **E_2_** | **Fol>14mm#** | **Total oocyte#** |
| **Mean** | -33.9 | -2.09 | 4.47 | -5.39 |
| **Median** | -26.5 | -14.2 | -8.3 | -7.14 |
| **Std. Deviation** | 23 | 48.8 | 48.2 | 32.2 |
| **Minimum** | -89.6 | -65.2 | -56.2 | -66.6 |
| **Maximum** | -5.2 | 136.3 | 180 | 60 |
| **25**  **Percentiles 50**  **75** | -46.9 | -42.3 | -25.9 | -30.3 |
|  | -26.4 | -14.2 | -8.3 | -7.14 |
|  | -17.1 | 23.7 | 33.3 | 16.6 |

**Supplementary Figure-1**


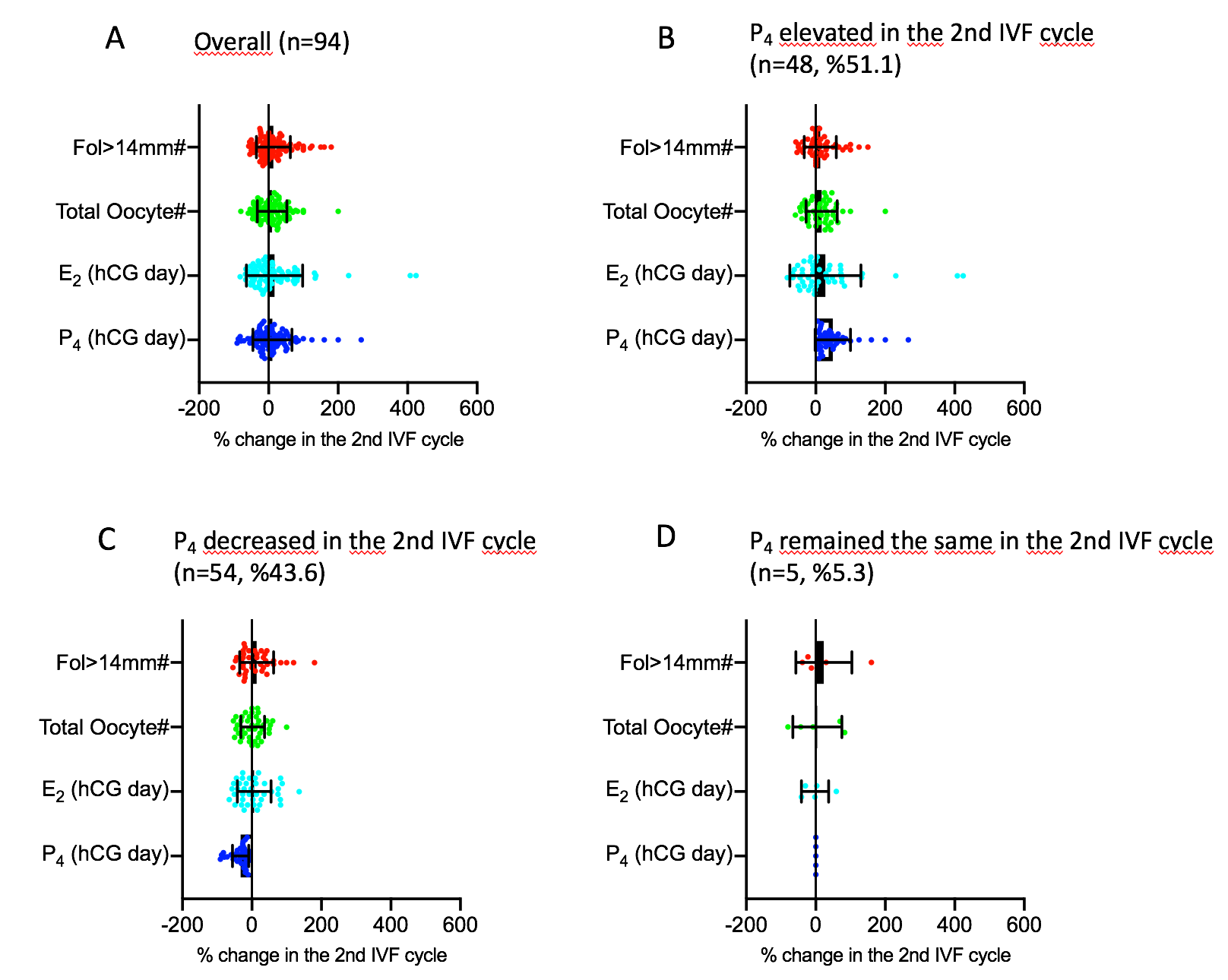


**Supplementary Figure-2**


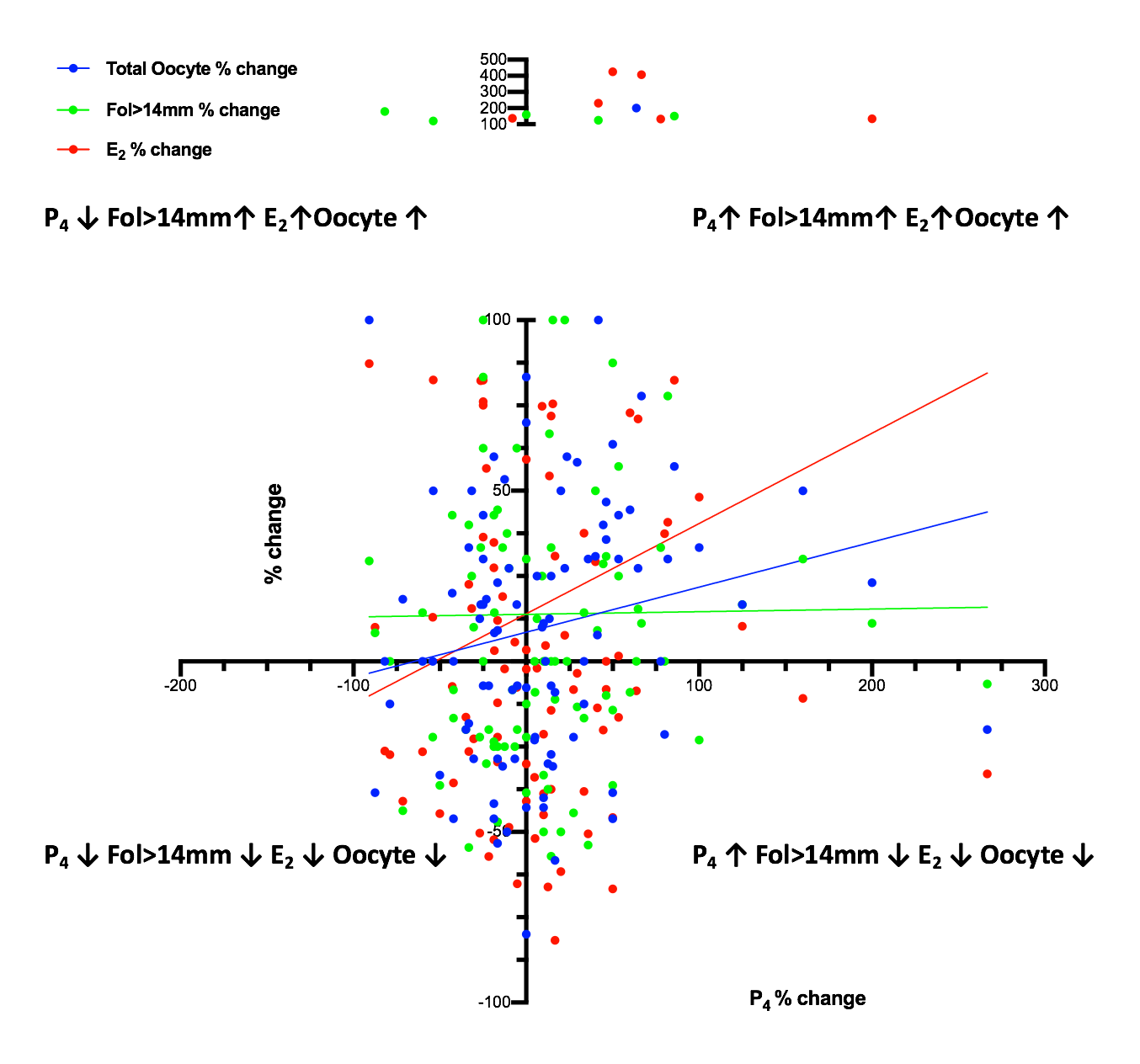

Supplement: Supplementary file 1 — Supplementary file1 [file 41598_2020_72597_MOESM1_ESM.docx]
